# Efficacy and Safety of Rituximab in Refractory CIDP With or Without IgG4 Autoantibodies (RECIPE): Protocol for a Double-Blind, Randomized, Placebo-Controlled Clinical Trial

**DOI:** 10.2196/17117

**Published:** 2020-04-01

**Authors:** Shinobu Shimizu, Masahiro Iijima, Yuki Fukami, Natsuko Tamura, Masahiro Nakatochi, Masahiko Ando, Ryoji Nishi, Haruki Koike, Kenichi Kaida, Michiaki Koga, Takashi Kanda, Hidenori Ogata, Jun-Ichi Kira, Masahiro Mori, Satoshi Kuwabara, Masahisa Katsuno

**Affiliations:** 1 Department of Advanced Medicine Nagoya University Hospital Nagoya Japan; 2 Department of Neurology Nagoya University Graduate School of Medicine Nagoya Japan; 3 Center for Integrated Medical Research Hiroshima University Hospital Hiroshima Japan; 4 Department of Nursing Nagoya University Graduate School of Medicine Nagoya Japan; 5 Department of Neurology Anti-aging and Vascular Medicine National Defense Medical College Tokorozawa Japan; 6 Department of Neurology and Clinical Neuroscience Yamaguchi University Graduate School of Medicine Ube Japan; 7 Department of Neurology Neurological Institute Graduate School of Medical Sciences, Kyushu University Fukuoka Japan; 8 Department of Neurology Graduate School of Medicine, Chiba University Chiba Japan

**Keywords:** chronic inflammatory demyelinating polyradiculoneuropathy, rituximab, immunoglobulin G4 autoantibodies, clinical study, partially randomized controlled trial

## Abstract

**Background:**

Chronic inflammatory demyelinating polyradiculoneuropathy (CIDP) is an immune-mediated peripheral neuropathy that is currently classified into several clinical subtypes, which are presumed to have different pathogenic mechanisms. Recently, studies identified a subgroup of patients with CIDP who were positive for IgG4 autoantibodies against paranodal proteins, such as neurofascin-155 and contactin-1, who respond poorly to first-line therapies for typical CIDP, including intravenous immunoglobulin therapy.

**Objective:**

This study aims to evaluate the efficacy and safety of intravenous rituximab according to IgG4 autoantibody status in patients with refractory CIDP.

**Methods:**

The Evaluation of the Efficacy and Safety of Rituximab in Refractory CIDP Patients with IgG4 Autoantibodies in the Exploratory Clinical (RECIPE) trial consists of 2 cohorts: a multicenter, placebo-controlled, randomized study cohort of 15 patients with IgG4 autoantibody-positive CIDP (rituximab:placebo = 2:1) and an open-label trial cohort of 10 patients with antibody-negative CIDP. The primary endpoint is improvement in functional outcome assessed using the adjusted Inflammatory Neuropathy Cause and Treatment Disability Scale score at 26, 38, or 52 weeks after the start of treatment with rituximab in patients with CIDP and anti-paranodal protein antibodies. Secondary outcome measures include grip strength, manual muscle testing sum scores, results of nerve conduction studies, and other functional scales.

**Results:**

We plan to enroll 25 cases for the full analysis set. Recruitment is ongoing, with 14 patients enrolled as of January 2020. Enrollment will close in September 2020, and the study is planned to end in December 2021.

**Conclusions:**

This randomized controlled trial will determine if rituximab is safe and effective in patients with anti-paranodal antibodies. An open-label study will provide additional data on the effects of rituximab in patients with antibody-negative CIDP. The results of the RECIPE trial are expected to provide evidence for the positioning of rituximab as a pathogenesis-based therapeutic for refractory CIDP.

**Trial Registration:**

ClinicalTrials.gov NCT03864185, https://clinicaltrials.gov/ct2/show/NCT03864185 ; The Japan Registry of Clinical Trials jRCT2041180037, https://jrct.niph.go.jp/en-latest-detail/jRCT2041180037

**International Registered Report Identifier (IRRID):**

DERR1-10.2196/17117

## Introduction

Chronic inflammatory demyelinating polyradiculoneuropathy (CIDP) is an acquired demyelinating peripheral neuropathy characterized by weakness and sensory disturbance in the extremities that develops over a period of at least 2 months. The current diagnostic criteria for CIDP were published by the European Federation of Neurological Societies/Peripheral Nerve Society (EFNS/PNS) and include clinical and electrophysiological findings with supporting features that strengthen the diagnosis [1-4]. The global prevalence of CIDP diagnosed using these criteria is around 3 per 100,000 population, and it is considered an orphan disease. However, the etiology of CIDP has not been fully clarified, and a biomarker reflecting its pathogenesis is lacking, so the exact prevalence remains uncertain [5-10]. One of the reasons why the etiology remains unclear might be the diagnostic vulnerability of CIDP. Although the EFNS/PNS criteria identify the typical form and 5 atypical forms by phenotype, there is no biomarker that explains each phenotype. Moreover, although a wide range of cellular and humoral autoimmunity factors are associated with CIDP onset, there is no specific immune mechanism that corresponds to each atypical CIDP subtype, although macrophages are assumed to be the main effector in typical CIDP.

Autoantibodies targeting myelin components, inflammatory cytokines, and the complement pathway are assumed to be the humoral factors modifying immune status in CIDP. Although many autoantibodies against various myelin antigens, such as P0, PMP22, Cx32, beta-tubulin, LM1, and sulfatide, have been investigated, none has been implicated in the pathogenesis of CIDP [11,12]. Autoantibodies that target contactin-1 (CNTN1), neurofascin-155 (NF155), and contactin-associated protein 1 (CASPR 1) have been identified in patients with slowly progressing CIDP phenotypes. The anti-CNTN1 antibody has been identified in only a small percentage of patients with CIDP, the anti-NF155 antibody has been identified in approximately 10%, and the anti-CASPR 1 antibody appears to be very rare [13-15]. Most anti-NF155 antibody–positive patients tend to develop tremor and sensory ataxia with distal weakness and muscle atrophy. They also have extremely high cerebrospinal fluid protein levels (usually >200 mg/dL), and gadolinium-enhanced magnetic resonance imaging shows severe swelling of the nerve roots or plexus. CIDP with anti-NF155 and anti-CNTN1 antibodies have several clinically similar characteristics. However, compared with anti-NF155–positive patients, anti-CNTN1–positive patients are relatively older at disease onset and have more rapid disease development. Approximately 70% of patients with these immunoglobulin (Ig)G4 autoantibodies are resistant to first-line therapies, such as intravenous immunoglobulin (IVIg) [13,14,16-18]. IVIg has immunomodulatory activity via several mechanisms, including immune regulation by macrophages or antigen-presenting cells via Fc receptors and idiotypic antibodies, suppression of activating cytokines, and the complement pathway. However, it would be inappropriate to induce Igs to inhibit complement-associated pathogenesis in patients with IgG4 autoantibody–positive CIDP because IgG4 lacks a complement-binding site and has no or limited ability to activate the classical complement pathway. Moreover, IVIg has been found ineffective as initial treatment for IgG4 autoantibody–positive CIDP. Long-term use of corticosteroids is challenging because of adverse reactions, including susceptibility to infection, osteoporosis, abnormal glucose tolerance, hyperlipidemia, and, possibly, psychiatric symptoms and insomnia. Plasmapheresis is a first- or second-line therapeutic strategy for CIDP but is difficult to implement as maintenance therapy because of high costs and safety issues associated with vascular access and fluctuations in plasma circulation. Therefore, novel therapeutics that can achieve long-term suppression of pathogenic antibodies are needed for IgG4 autoantibody–positive CIDP. Rituximab, a chimeric anti-CD20 monoclonal antibody, selectively depletes B cells and might suppress production of antibodies by inhibiting their differentiation into plasma cells. Also, some case reports suggest that rituximab is effective in refractory CIDP [19-29]. However, all these reports were retrospective and uncontrolled, and there is little evidence to suggest that rituximab is effective for IgG4 autoantibody–positive CIDP [30-32]. Moreover, the efficacy of rituximab has been proven in other IgG4 diseases such as anti-muscle-specific tyrosine kinase (MuSK) myasthenia gravis [33,34].

Therefore, we planned this prospective clinical trial to investigate the efficacy of rituximab in refractory IgG4 autoantibody–positive CIDP by comparing with placebo in IgG4 autoantibody–positive patients and with rituximab in IgG4 autoantibody–negative patients as a reference. This investigator-initiated clinical trial has been named “The Evaluation of Efficacy and Safety of Rituximab (Genetical Recombination) in Refractory Chronic Inflammatory Demyelinating Polyneuropathy (CIDP) Patients with Immunoglobulin G4 (IgG4) Autoantibodies in the Exploratory Clinical Trial” (RECIPE trial). The design of the trial was developed in consultation with and approved by the Pharmaceuticals and Medical Device Agency (PMDA), which is responsible for reviewing new pharmaceuticals in Japan.

Here, we provide the detailed design of this investigator-initiated clinical trial in refractory CIDP patients with or without IgG4 autoantibodies as an exploratory study in Japan (the RECIPE trial).

## Methods

### Objectives and Endpoints

**Figure 1:**
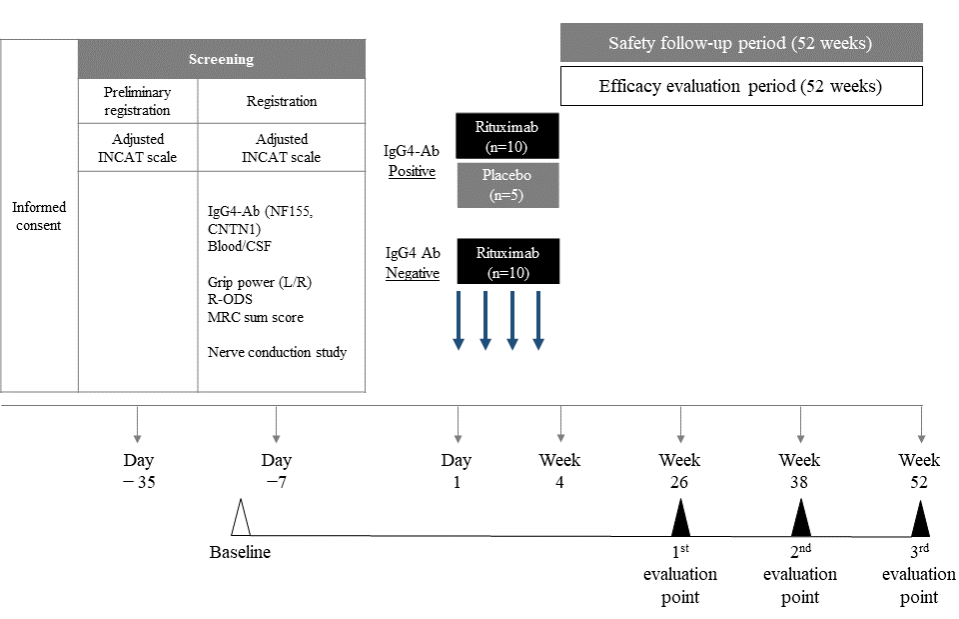
Measurement schedule. CNTN!: Contactin-1; CSF: cerebrospinal fluid; IgG4-Ab: immunoglobulin G4 antibodies; INCAT: Inflammatory Neuropathy Cause and Treatment; MRC: Medical Research Council; NF155: Neurofascin-155; R-ODS: Rasch-built Overall Disability Scale.

The primary objectives of the RECIPE trial are to evaluate the efficacy and safety of intravenous rituximab in patients with refractory CIDP according to their IgG4 autoantibody status. This trial consists of a multicenter, placebo-controlled, randomized, double-blind, parallel-group, comparative study in 15 patients with CIDP and IgG4 autoantibodies (CNTN1 or NF155) who will be allocated to a rituximab group (n=10) or a placebo group (n=5) and an open-label study in 10 patients with IgG4 autoantibody–negative CIDP. The outline of this study is shown in Figure 1.

The primary endpoint is improvement in the adjusted Inflammatory Neuropathy Cause and Treatment (INCAT) Disability Scale score at 26, 38, or 52 weeks after the start of treatment. Efficacy is based on the effectiveness rate, which is the proportion of patients who achieve the primary endpoint of ≥1-point improvement in the INCAT Disability Scale score compared with baseline. Our specific hypothesis is that the effectiveness rate will exceed a prespecified threshold of 15% in patents with IgG4 autoantibody–positive CIDP in the rituximab-treated group. The data from patients with IgG4 autoantibody–negative CIDP will serve as a reference.

### Study Population

The RECIPE trial will investigate the efficacy of rituximab in patients with refractory IgG4 autoantibody–positive and IgG4 autoantibody–negative CIDP. Anti-NF155 and anti-CNTN1 autoantibody status will be confirmed by enzyme-linked immunosorbent assay as described in the literature [17,35].

### Inclusion Criteria

Patients who meet the following criteria are eligible to participate:

1. CIDP diagnosed according to the modified EFNS/PNS criteria [1-4] prior to enrollment
2. Positive or negative serum IgG4 autoantibody (CNTN1 or NF155) status confirmed prior to enrollment
3. CIDP refractory to treatment with corticosteroids for 12 weeks and IVIg for 8 weeks prior to enrollment or unable to receive or continue treatment with corticosteroids and IVIg
4. Total adjusted INCAT Disability Scale scores of 2 to 8 at both preliminary enrollment and enrollment, with no decrease in total score between preliminary enrollment and enrollment
5. Age ≥12 years
6. Able to provide voluntary written consent after receiving adequate information about the study (for children aged 12 to 15 years, consent will be obtained from an acceptable representative, and informed assent will be obtained from the patient)

### Exclusion Criteria

Patients are excluded for any of the following reasons:

1. Any differential diagnosis defined in the modified EFNS/PNS diagnostic criteria [1-4]
2. Initiation or an increased dose of corticosteroids within 12 weeks before enrollment
3. Initiation or an increased dose of IVIg within 8 weeks before enrollment
4. Plasmapheresis within 8 weeks before enrollment or disease refractory to 8 weeks of plasma exchange or double-filtration plasmapheresis
5. Initiation or an increased dose of an immunosuppressant (ie, azathioprine, cyclophosphamide, cyclosporine, mycophenolate mofetil, interferon-alpha, interferon-beta, etanercept, methotrexate, mitoxantrone, alemtuzumab, cladribine, tacrolimus, or fingolimod) within 12 weeks before enrollment
6. History of hematopoietic stem cell transplantation
7. Previous treatment with rituximab
8. Participation in another clinical study within 3 months before enrollment or current participation in another study
9. Poorly controlled diabetes (hemoglobin A_1c_ ≥7%)
10. Suspected or confirmed infection requiring systemic treatment with an antimicrobial, antifungal, or antiviral agent at enrollment
11. Positive for hepatitis B surface antigen or antibody, hepatitis B core antibody, hepatitis C virus antibody, or HIV or human T-cell lymphotropic virus-1 antibody positivity at enrollment
12. Leukopenia (<2000/mm^3^), neutropenia (<1000/mm^3^), or lymphopenia (<500/mm^3^) at enrollment
13. History of severe hypersensitivity or anaphylactic reaction to any of the ingredients in the investigational drug or murine protein-containing products
14. Severe comorbidity (eg, hepatic, renal, cardiac, lung, hematologic, or brain disease)
15. Pregnancy or potential for pregnancy, lactation, or unwillingness to use contraception during the study period
16. Deemed unsuitable for participation by an investigator or sub-investigator

Regarding item 11, patients with positive hepatitis B surface antibody or core antibody can be enrolled when a hepatitis B virus-DNA test is negative (below the limit of detection), and hepatitis B virus-DNA and aspartate/alanine transaminase levels are monitored at fixed intervals.

### Registration and Randomization

The investigators obtain written informed consent from all patients who are potentially eligible for the study. After confirmation of inclusion criteria 4 to 6 and exclusion criteria 1, 6, 7, and 13 to 16, patients are preliminarily enrolled via an electronic data capture system (Viedoc™, PCG Solutions Ab. Uppsala, Sweden). At the second screening, the investigators check all the inclusion and exclusion criteria. Patients confirmed to have IgG4 autoantibody–positive CIDP are randomly assigned to the rituximab group or the placebo group at a ratio of 2:1 using the stratified permuted block method according to their adjusted INCAT Disability Scale score (2 to 4 or 5 to 8). Patients with IgG4 autoantibody–negative CIDP are automatically allocated to the rituximab group.

### Investigational Treatment

Rituximab 375 mg/m^2^ or placebo is administered intravenously once weekly for 4 weeks. This is the same dosage as used in previous studies involving CIDP patients treated with rituximab [20-25,27,30,31]. This dosage is also the same as that used in a phase I/II trial that showed peripheral B cells (CD20-positive) were adequately decreased in patients with B-cell non-Hodgkin's lymphoma or nephrotic syndrome. The time taken for these cells to return to their baseline level was at least 5-7 months in that study.

Hematopoietic stem cell transplantation, plasma exchange therapy (simple plasma exchange or double membrane filtration), and any drug that could affect immune status is prohibited during the trial in view of their potential to affect the efficacy of rituximab. For the same reason, starting or increasing the dose of corticosteroid, IVIg, or immunosuppressant therapy is not permitted for the duration of the study.

### Primary Endpoint

All participants are followed for 52 weeks after the first dose of investigational medication (Table 1). All data are collected via an electronic data capture system and checked according to the data management and monitoring plan. Adjusted INCAT Disability Scale score is used to evaluate lower (gait) and upper extremity disorders; this tool has been used as an efficacy endpoint in clinical investigations of IVIg as a treatment for CIDP [36,37]. A change of ≥1 point in any of the items on this scale is considered clinically significant in terms of the ability to perform activities of daily living. Therefore, we selected a change of ≥1 point in the adjusted INCAT Disability Scale score as the primary endpoint. The primary analysis will compare the adjusted INCAT Disability Scale scores recorded before treatment (at enrollment) and those recorded at week 26, 38, or 52.

**Table 1:** Protocol for data collection from each patient enrolled in the RECIPE trial.

Visit	Screening							
	Preliminary registration	Registration	Administration	Weeks 1, 2, and 3	Week 4	Week 12	Week 26	Wek 38	Week 52 or withdrawal
Allowance (days)	up to –35	–34 to –7	–3	–3	±3	±14	±14	±14	±28
Informed consent									
Administration of investigational drug			x	x					
Hospitalization			x	x	x				
Basic information (eg, birth date, sex, body weight)	x	x							
Eligibility	x	x							
Pregnancy test	x	x							
Vital signs^a^		x	x	x	x	x	x	x	x
Oxygen saturation			x^b^	x^b^	x				
Hematology^c^		x	x	x	x	x	x	x	x
Blood chemistry^d^		x	x	x	x	x	x	x	x
Other blood tests^e^		x							
Urinalysis^f^		x	x	x	x	x	x	x	x
Chest X-ray		x				x	x		x
Adjusted INCAT^g^ Disability Scale	x^h^	x^h^			x	x	x	x	x
Grip power (vigorimeter) Left/Right		x			x	x	x	x	x
Rasch-built Overall Disability Scale		x			x	x	x	x	x
MRC-SS^i^		x			x	x	x	x	x
Nerve conduction study		x			x		x	x	x
CSF^j^ protein		x					x	x	x
Serum autoantibody^k^	x^l^						x	x	x
Serum neurofilament light		x					x	x	x
B cell/T cell (whole blood, %)^m^			x		x	x	x	x	x
HACA^m,n^			x				x		x
Pharmacokinetics^m^		x	x^o^	x^o^	x	x	x	x	x
Concomitant medication	x	x	x	x	x	x	x	x	x
Adverse event(s)			x	x	x	x	x	x	x

^a^Blood pressure, pulse rate, and body temperature.

^b^Measured within 30 minutes before the start of dose administration; immediately before a change in dosing rate, dose interruption, or dose reduction; before dose administration is resumed; and within 10 minutes and 1 hour after the end dose administration.

^c^Red blood cell count, hemoglobin, hematocrit, white blood cell count, differential white blood count (basophils, eosinophils, neutrophils, lymphocytes, and monocytes), and platelet count.

^d^Blood urea nitrogen, creatinine, lactate dehydrogenase, aspartate/alkaline transaminase, alkaline phosphatase, gamma glutamyl transpeptidase, total bilirubin, direct bilirubin, creatine kinase, and C-reactive protein.

^e^Glycated hemoglobin, hepatitis B surface antigen, hepatitis B surface antibody, hepatitis B virus-DNA if needed, hepatitis B core antibody, hepatitis C virus antibody, HIV antibody, and human T-cell lymphotropic virus-1.

^f^Urine protein, urine occult blood, urine glucose, and pH.

^g^INCAT: Inflammatory Neuropathy Cause and Treatment.

^h^If more than 28 days has passed between provisional enrollment and enrollment.

^i^MRC-SS: Medical Research Council Sum-Score.

^j^CSF: cerebrospinal fluid.

^k^Further measurement is not required if not detected at screening.

^l^Measured only once during the screening period.

^m^Test results are not disclosed during the study period.

^n^HACA: human anti-chimeric antibody.

^o^Blood is collected within 15 minutes before the start and after the end of dose administration.

### Secondary, Exploratory, and Safety Endpoints

Secondary endpoints include grip strength, as measured using a vigorimeter (left/right); the Rasch-built Overall Disability Scale; Medical Research Council sum score; median, ulnar, tibial, and peroneal motor nerve conduction studies, including motor nerve conduction velocity and distal and proximal latency, amplitude, and duration of the compound muscle action potential after distal and proximal stimulation; protein levels in the cerebrospinal fluid; B cell (CD19-positive and CD20-positive) counts and T-cell (CD3-positive, CD4-positive, and CD8-positive) counts; expression of human anti-chimeric antibodies; and serum rituximab level.

The exploratory endpoints include serum antibody titers of IgG4, serum antibody titers of anti-CNTN1 and anti-NF155 IgG subclasses, and serum neurofilament level.

The safety endpoints include adverse events and vital signs and laboratory tests.

### Sample Size

The trial includes patients with CIDP that is refractory to both IVIg and corticosteroids or patients who are unable to receive or continue these therapies. Most eligible patients would be unlikely to achieve an improvement of ≥1 point on the adjusted INCAT Disability Scale even if conventional therapies were continued. An immunosuppressant (eg, cyclophosphamide, azathioprine, or cyclosporine) is generally considered to be an effective second-line treatment in about 30% of patients with refractory CIDP [19]. However, no superiority has been demonstrated for any specific agent, and no immunosuppressant is approved for CIDP in Japan. Currently, no feasible treatment approach can be recommended for patients enrolled in this study. Therefore, the threshold effectiveness rate for the proportion of patients achieving the primary endpoint of ≥1 point improvement in the adjusted INCAT Disability Scale score was conservatively assumed to be 15%, and the clinically expected effectiveness rate was assumed to be 60% in patients with IgG4 autoantibody–positive CIDP in the rituximab group. Using two-sided testing with *P*<0.05 regarded as statistically significant, we estimated that 8 cases would be needed to achieve 80% power. Accordingly, 10 patients with IgG4 autoantibody–positive CIDP will be enrolled for the rituximab group, allowing for a 20% dropout rate.

The PMDA suggested inclusion of a placebo control group for the patients who are IgG4 autoantibody–positive. We agreed to include a placebo group of 5 patients for reference purposes rather than for strict statistical comparison, given the rarity of CIDP and feasibility considerations. The placebo group has been designated as a reference group to confirm that the estimated values do not deviate markedly from the defined thresholds. Patients with IgG4 autoantibody–negative CIDP are included in another reference group to compare their response to rituximab treatment with that of patients with IgG4 autoantibody–positive CIDP. This reference group will consist of 10 patients, which is the same as the number of patients with IgG4 autoantibody–positive CIDP.

### Statistical Analysis

All statistical analyses will be performed on an intention-to-treat basis. The primary analysis will compare the adjusted INCAT Disability Scale scores recorded before treatment (at enrollment) with those recorded at 26, 38, or 52 weeks after the start of treatment. We will calculate the proportion and 95% CI of IgG4 autoantibody–positive patients in the rituximab group who achieve an improvement of ≥1 point at any of evaluation point after 26 weeks, from baseline. The 95% CI will be calculated using the Clopper-Pearson method. Continuous variables will be analyzed using descriptive statistics, and categorical variables will be calculated as the frequency and proportion. All statistical analyses will be performed using SAS version 9.4 (SAS Institute Inc, Cary, NC). All statistical tests will be two-sided. *P* values <0.05 will be considered statistically significant.

### Monitoring and Auditing

Monitoring and auditing will include systematic independent examination according to the study protocol, applicable regulatory requirements, and standard operating procedures.

## Results

### Ethics Approval, Trial Registration, and Current Enrollment Status

The study protocol complies with the Declaration of Helsinki [38] and the Pharmaceutical Affairs Act in Japan. This protocol was also approved by the institutional review boards at the following sites: Nagoya University Hospital (No. 302010), Chiba University Hospital (No. 030033), Yamaguchi University Hospital (No. 201901), and Kyushu University Hospital (No. 2018312).

The necessary information for the RECIPE trial has been uploaded to ClinicalTrials.gov (NCT03864185, registered March 6, 2019) and the Japan Registry of Clinical Trials (jRC2041180037, registered January 31, 2019).

The first patient completed registration in April 2019 and received an investigational treatment in May 2019. Recruitment of patients for the RECIPE trial is ongoing at the four participating hospitals. As of January 2020, 14 cases have been enrolled. The targeted accrual is 25 cases for the full analysis set. Enrollment will close in September 2020, and the study is scheduled to end in December 2021.

## Discussion

### Overview

There have been some reports on the epidemiology of IgG4 antibody–positive CIDP [14,16,17]. This phenotypic subtype presents as subacute or slowly progressive disease. The initial disability tends to be distal acquired demyelinating symmetric neuropathy, which sometimes progresses to the typical CIDP phenotype. IgG4 antibody–positive CIDP is also characterized by gait disturbance with sensory ataxia and fine tremor of the hands. The protein level in cerebrospinal fluid is markedly higher in IgG4 antibody–positive CIDP than in typical CIDP. Furthermore, a relatively large proportion of patients with antibody–positive CIDP present at a younger age. However, they are resistant to conventional therapies, such as IVIg and corticosteroids. Therefore, new therapies that can inhibit production of pathogenic antibodies in the long term are essential.

Rituximab can be expected to be effective in cases of refractory CIDP [19-32]. Some of the phenotypic characteristics, for example onset time, are different from those in anti-CNTN1–positive patients and anti-NF155 patients; however, patients with IgG4 autoantibody–positive CIDP have several clinically common characteristics. Given its mechanism of action, rituximab is likely to be effective in patients with IgG4 autoantibody (anti-CNTN1 and anti-NF155)–positive CIDP. Accordingly, we are planning to develop rituximab for use in these patients and present here the protocol for the exploratory study intended to secure approval of rituximab in Japan. We have attended a consultation meeting with the PMDA, which has agreed to the development strategy and initial design of the RECIPE trial.

### Conclusion

This article has described the design and protocol being used in the RECIPE trial. We outlined the characteristics of patients with IgG4 autoantibody–positive CIDP and discussed some critical considerations for these patients. The RECIPE trial is the first randomized controlled trial of rituximab for IgG4 autoantibody–positive CIDP. It is anticipated that the results will lead to a pathogenesis-oriented therapeutic strategy that can target specific phenotypes of CIDP and confirm the efficacy of rituximab in refractory IgG4 autoantibody–negative CIDP.

## Abbreviations

CASPR 1: contactin-associated protein 1.
CIDP: chronic inflammatory demyelinating polyradiculoneuropathy.
CNTN1: contactin-1.
EFNS/PNS: European Federation of Neurological Societies/Peripheral Nerve Society.
Ig: immunoglobulin.
INCAT: Inflammatory Neuropathy Cause and Treatment.
IVIg: intravenous immunoglobulin.
MuSK: muscle-specific tyrosine kinase.
NF155: neurofascin-155.
PMDA: Pharmaceuticals and Medical Device Agency.
